# Callosal Angle Sub-Score of the Radscale in Patients with Idiopathic Normal Pressure Hydrocephalus Is Associated with Positive Tap Test Response

**DOI:** 10.3390/jcm11102898

**Published:** 2022-05-20

**Authors:** Efstratios-Stylianos Pyrgelis, George P. Paraskevas, Vasilios C. Constantinides, Fotini Boufidou, Georgios Velonakis, Leonidas Stefanis, Elisabeth Kapaki

**Affiliations:** 1 1st Department of Neurology, School of Medicine, Eginition Hospital, National and Kapodistrian University of Athens, 74 Vass. Sophias Ave., 11528 Athens, Greece; vassilis.kon@hotmail.com (V.C.C.); lstefanis@med.uoa.gr (L.S.); ekapaki@med.uoa.gr (E.K.); 2 Neurochemistry and Biological Markers Unit, 1st Department of Neurology, School of Medicine, Eginition Hospital, National and Kapodistrian University of Athens, 74 Vass. Sophias Ave., 11528 Athens, Greece; geoprskvs44@gmail.com (G.P.P.); fboufidou@med.uoa.gr (F.B.); 3 2nd Department of Neurology, School of Medicine, “Attikon” University General Hospital, National and Kapodistrian University of Athens, Rimini 1, Chaidari, 12462 Athens, Greece; 4 Research Unit of Radiology, 2nd Department of Radiology, Medical School, “Attikon” University General Hospital, National and Kapodistrian University of Athens, Rimini 1, Chaidari, 12462 Athens, Greece; giorvelonakis@gmail.com

**Keywords:** normal pressure hydrocephalus, Radscale, MRI, tap test

## Abstract

The aim of the present study was the implementation of the composite imaging “Radscale” in patients with idiopathic normal pressure hydrocephalus (iNPH) and the evaluation of its score, as well as absolute stroke volume and peak flow velocity of cerebrospinal fluid (CSF) in aqueduct as indicators of a positive response following a tap test. Forty-five patients with iNPH were included. Clinical evaluation involved the 10 m timed walk test before and every 24 h for 3 consecutive days after evacuative lumbar puncture (LP). Neuropsychological evaluation comprised a mini mental state examination (MMSE), frontal assessment battery (FAB), 5-word test (5WT) and CLOX drawing test 1 and 2, which were carried out before and 48 h after LP. The tap test’s response was defined as a ≥20% improvement in gait and/or a ≥10% improvement in neuropsychological tests. All scores of neuropsychological and clinical variables, except for immediate 5WT and CLOX-1, differed significantly before and 48 h after LP. Improvement in time and steps of a 10 m timed walk test differed significantly between female and male patients. Out of 45 total patients, 19 were tap test responders and 26 non-responders. The total score of Radscale and CSF flow parameters did not differ between responders and non-responders. However, “Callosal angle” sub-score differed significantly between these two groups. A greater “callosal angle” sub-score, meaning more acute callosal angle, was associated with a positive tap test response, rendering it a useful measurement in the stratification of iNPH patients that will potentially respond to CSF shunting.

## 1. Introduction

Normal pressure hydrocephalus (NPH) is a form of communicating hydrocephalus, characterized by ventriculomegaly and normal intraventricular cerebrospinal fluid (CSF) pressure that occurs more frequently in elderly patients. It is distinguished into idiopathic NPH (iNPH) and secondary NPH, as a result of subarachnoid hemorrhage, traumatic brain injury, infection, tumor, etc. [1,2,3]. Idiopathic NPH was first described in 1965 by Adams and colleagues as a triad of gait disorder, cognitive impairment (of the subcortical type) and urinary incontinence (Hakim’s triad) [4,5]. The incidence of iNPH in various studies ranges from 1.8 to 7.3/100,000, while its frequency increases with age [6]. Early diagnosis is of paramount importance, since iNPH could be a potentially reversible cause of dementia [2]. Ventriculoperitoneal (VP) shunt or third ventriculostomy are the surgical procedures used in patients predicted to respond [7,8]. One of the most well-established diagnostic tests and at the same time a prognostic factor of successful response to shunt surgery is the removal of 30–50 mL of the CSF and the consequent evaluation of motor and cognitive improvement after the lumbar puncture (LP), the so-called “tap test” [3,9].

According to the third edition of guidelines for the management of iNPH endorsed by the Japanese society of NPH, the diagnosis of iNPH requires concrete clinical and imaging criteria [10,11]. Thus, brain imaging in patients with iNPH is essential to diagnosis. However, many of the individual imaging parameters described in various studies lack adequate sensitivity and specificity [12]. To this end, a structured scale for the overall evaluation of imaging data has been proposed by Kockum et al., in 2015 and 2018, the so-called Radscale [13,14]. It is a calibrated imaging scale, consisting of morphological features of iNPH, and is thought to be capable of assessing the severity and possibly the effectiveness of the therapeutic intervention. It can be implemented on both computed tomography (CT) and magnetic resonance imaging (MRI). MRI has the advantage of better revealing periventricular white matter changes, as it has superior soft tissue contrast [15]. Brain MRI for iNPH can also include a phase-contrast MRI CSF flow study that can demonstrate CSF pulsatile flow during the cardiac cycle, by means of stroke volume and peak flow velocity [16].

The purpose of this study was the implementation of Radscale on MRI in patients with iNPH, the comparison of its total score and the score of its individual parameters between tap test responders and non-responders, as well as any possible associations among CSF stroke volume and peak flow velocity within these groups.

## 2. Materials and Methods

### 2.1. Study Population

A total of 45 subjects were included in the study. All subjects were recruited prospectively during the years 2019–2020 presenting to the 1st Department of Neurology of the National and Kapodistrian University of Athens at Eginition Hospital.

For inclusion in the study, patients had to receive the diagnosis of probable or possible iNPH according to the recent Guidelines for Management of Idiopathic Normal Pres-sure Hydrocephalus [10,11]. We have additionally included patients with Evan’s Index (EI) between 0.25 and 0.3 (four in total number), who fulfilled the rest clinical and imaging criteria with the purpose of not excluding patients with an EI sub-score 1 in the Radscale. Patients with a history of stroke or systemic health issues with potential cerebral impairment were excluded.

All patients underwent extensive clinical, biochemical, immunological and endocrine examination, in order to exclude other (neurological or systemic) causes of gait disturbances and/or cognitive impairment.

The iNPH grading scale was used as a quantitative scale to separately assess the severity of each of the 3 symptoms of iNPH (cognitive impairment, gait disturbance and urinary disturbance) [17].

### 2.2. Ethical Issues

The study was in accordance with the ethical guidelines of the Declaration of Helsinki and had the approval of the local Ethical and Deontology committee of our hospital. All subjects and/or relatives gave informed consent for inclusion in the study.

### 2.3. Magnetic Resonance Imaging

MRI examinations were performed once, before LP, on a 3 Tesla Achieva TX Philips manufactured MRI scanner (Philips, Best, the Netherlands); MRI protocol included 3D T2 Flair, 3D T1, Ax PD/T2, SWI, Ax DTI and phase-contrast flowmetry analysis.

**Figure 1:**
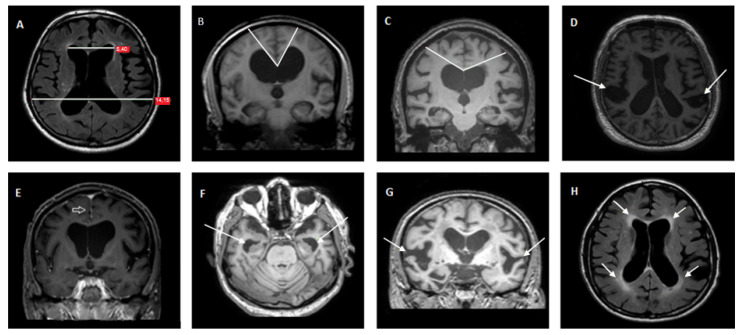
Brain T1-weighted (B-G) and FLAIR (**A**,**H**) MRI images of patients of this study. Arrows are pointing to the areas of interest of each imaging parameter. Evan’s Index measurement (graded as 0 if <0.25, 1 if 0.25–0.3 and 2 if >0.3) (**A**). Callosal angle <60° in a coronal slice (callosal angle is graded as 0 if >90°, 1 if 90° to >60° and 2 if ≤60°) of a tap test responder (**B**) and >90° in a coronal slice of a non-responder (**C**). Focally enlarged sulci in a transverse slice (graded as 0 if not present, 1 if present) (**D**). Narrow sulci in a coronal slice (parameter graded as 0 for normal, 1 for parafalcine and 2 for vertex) (**E**). Dilated temporal horns in a transverse slice (graded as 0 if <4 mm, 1 if 4 to <6 mm and 2 if ≥6 mm) (**F**). Dilated Sylvian fissures in a coronal slice (graded as 0 if normal, 2 if enlarged) (**G**). Diffuse periventricular hyper intensity in a transverse slice (graded as 0 if not present, 1 for frontal horn caps and 2 for confluent areas) (**H**).

For the implementation of the Radscale on MRI, seven imaging parameters were measured (Figure 1): (1) Evans’ index representing the ratio of maximum width of the frontal horns of the lateral ventricles and the maximal internal diameter of the skull at the same level; (2) Callosal angle measured on a coronal image perpendicular to the anterior commissure–posterior commissure plane at the level of the posterior commissure; (3) focally dilated sulci recognized in coronal or transverse planes; (4) narrow parietal high-convexity and medial parafalcine sulci assessed in the transverse plane in the most superior slices and in the coronal plane; (5) the size (as a mean width of the right and left side) of temporal horns measured in the transverse plane; (6) dilated Sylvian fissures evaluated in the coronal plane; and (7) periventricular abnormal density around the lateral ventricles [14]. Implementation of Radscale was performed in a blind fashion with regard to clinical diagnosis by a neurologist with experience in neuroradiology. All measurements were performed on T1 sequences.

CSF flow dynamics were evaluated on phase contrast sequences oriented perpendicular to the aqueduct (repetition time (TR): 12 ms, time echo (TE): 7.5 ms, flip angle: 15, slice thickness 4 mm, matrix size 292 × 203 mm). The velocity encoding values were 9–20 cm/sec. Retrospective cardiac gating was performed. Regions of interest were drawn manually in the aqueduct. Peak flow velocity and absolute stroke volume were calculated using Philips Q-flow software (IntelliSpace Portal 9.0; Philips Medical Systems, Best, The Netherlands).

### 2.4. Clinical and Neuropsychological Evaluation

The LP was always performed in the morning, with the patient in the lateral decubitus position. The opening pressure of CSF was measured and patients with opening pressure higher than 20 cm H_2_O were excluded from the study. LP was performed using an 18- or 20-gauge spinal needle removing 30 mL (to 50 mL) of CSF. Clinical assessment included gait evaluation using 10 m timed walk test (measurement of time and steps demanded to walk a 10 m distance), which was repeated every 24 h for 3 days after LP [18].

Neuropsychological examination was performed in all subjects before and 48 h after LP with a battery of neuropsychological tests comprising: (a) the mini-mental state examination (MMSE) to evaluate global cognitive status [19]; (b) the frontal assessment battery (FAB) to evaluate executive functions [20]; (c) the 5-word immediate and delayed recall (5WT) to evaluate memory [21] and (d) the 15-point spontaneous and copy CLOX drawing (CLOX1 and 2, respectively) as a sensitive test to executive control (CLOX1) vs. non-executive constructional failure (CLOX2) [22]. Clinical and neuropsychological assessment was performed by the same evaluator, a neurologist with experience in this field.

Response to the tap test was defined based on the following two criteria: (a) a ≥ 20% improvement in time or steps of the 10 m timed walk test and/or (b) a ≥ 10% improvement in at least MMSE and FAB. Based on these criteria, patients were divided into responders and non-responders [18,23].

### 2.5. Statistical Analysis

All numerical data were tested for normality and homogeneity of variances by the Shapiro–Wilk’s and Brown–Forsyth tests, respectively. For the variables that did not have normal distributions and homogenous variances, we used nonparametric tests for statistical analysis. Wilcoxon Matched Pairs test was used to investigate differences in score of neuropsychological and gait tests before and after LP. Mann–Whitney U Test was used to investigate differences of mean value of total score and sub scores of Radscale among tap test responders and non-responders. Multivariate linear regression adjusted for age and gender was performed to assess the association between callosal angle and responder status. Categorical data were compared between groups by the χ^2^-test. Differences and correlations between demographic, clinical, neuropsychological and radiological variables were investigated using the Kruskal–Wallis test and Spearman Rank correlation coefficient, followed by Bonferroni correction for multiple comparisons. All tests were performed using IBM SPSS Statistics^®^ version 23.0.0.0 (SPSS Inc., Chicago, IL, USA, 2013). All graphs were designed using GraphPad Prism^®^, version 8.43 (GraphPad Software Inc., La Jolla, CA, USA, 2020).

## 3. Results

**Figure 2:**
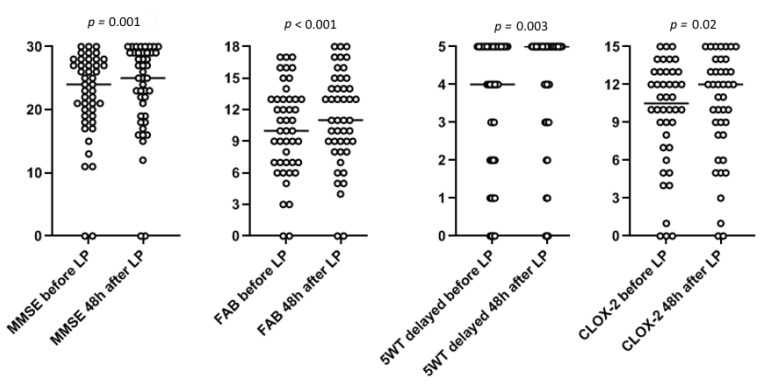
Change in the individual neuropsychological test scores for the whole group of patients before and 48 h after lumbar puncture. The horizontal bars represent median values of MMSE, FAB, 5-word test delayed recall and CLOX-2, respectively.

Out of the 45 patients recruited in the present study, 28 were males and 17 females. Their mean age was 74.4 years. Patients’ median values for iNPH grading scale score and disease duration were 6 points and 24 months, respectively. All scores of neuropsychological and clinical tests differed significantly before and 48 h after LP except for scores of immediate 5WT and CLOX-1 test (Table 1, Figure 2).

**Figure 3:**
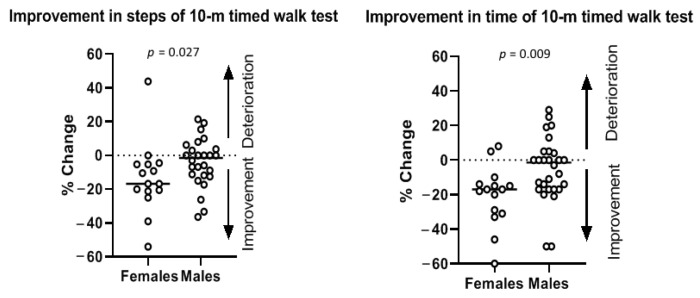
Improvement in steps (*p* = 0.027) and in time (*p* = 0.009) of 10 m timed walk test differed significantly between female and male patients with iNPH. The horizontal bars represent the median change in steps of 10 m timed walk test during tap test among male and female patients.

Improvement in time (*p* = 0.009) and steps of 10 m timed walk test (*p* = 0.027) differed significantly between female and male patients with iNPH (Figure 3), with women having greater percentile kinetic improvement than men in this particular test.

Patients were divided in two sub-groups, responders and non-responders, according to their tap test performance, as described above. Demographic and radiological characteristics of tap test responders and non-responders are shown in Table 2. Clinical and neuropsychological performance of responders and non-responders before and after LP are shown in Table 3, Table 4 and  Table S1.

**Figure 4:**
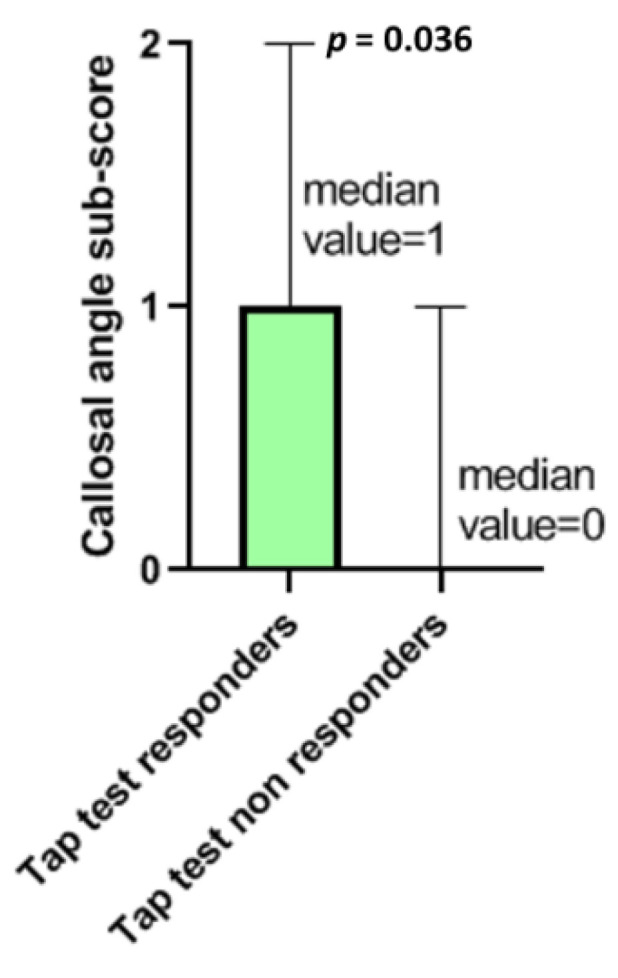
“Callosal angle” sub-score differed significantly between tap test responders and non-responders (*p* = 0.03). The median values and the range of “callosal angle” sub-sore values in the two groups are presented in this graph.

“Callosal angle” sub-score differed significantly between responders and non-responders, with the responders having more acute “callosal angle” (*p* = 0.036, with a moderate effect size r = 0.312) (Table 2, Figure 4). This association between higher callosal angle sub-score and positive tap test responder status remained significant even after adjusting for age and gender (β: 0.303, C.I. 95%: [0.013; 0.554], *p* = 0.041).

None of the other variants (including CSF flow measures, total Radscale and other sub-scores of the Radscale) differed significantly between responders and non-responders. Significant positive correlations were observed between various variables (Supplementary Figure S1); however, only the positive correlation of absolute stroke volume with peak flow velocity of CSF remained statistically significant (spearman R = 0.873, *p* = 0.012), when Bonferroni correction for multiple comparisons was applied (Supplementary Figure S2).

## 4. Discussion

In the present study, we implemented the newly proposed Radscale for patients with iNPH, previously applied on CTs, on patients MRIs and evaluated the total and individual sub-scores of its seven parameters, combined with other MRI measures, as indicators of the kinetic and neuropsychological response to evacuating LP.

MRI has advantages compared to CT in the evaluation of iNPH. Ref. [15] MRI, besides avoiding exposure to ionizing radiation, has also the advantages of being more accurate in evaluating white matter lesions, revealing accompanying pathologies and better detecting NPH typical signs of possible prognostic value [15,24], although every single imaging marker lacks adequate sensitivity and specificity. Additionally, CSF stroke volume and peak flow velocity can be measured using phase-contrast MRI throughout the cardiac cycle.

Current guidelines for iNPH use EI > 0.3 as a measurement for ventriculomegaly [10,11,23]; however, there are controversies in the use of this index and how it correlates with actual ventricles volume [25]. It has been reported that there are iNPH patients with EI lower than 0.3 [11]. In such a patient’s case, there was also a significant improvement after CSF shunting [26]. Thus, considering that the purpose of the present study was the association between Radscale total score, individual sub-scores and tap test responsiveness, we did not exclude patients with EI between 0.25 and 0.3, since they fulfilled the rest strict requirements described in the materials and methods section.

The tap test is the most well-established procedure for the prediction of patients that will possibly improve following ventriculo-peritoneal shunting. Besides being an invasive procedure, it also has low negative prognostic value. It also requires pre and post-test evaluation, which are not universally standardized while there is no gold standard on how to define positive tap test response in the current literature. Despite all of the above, it is widely used as an assessment tool of iNPH symptoms’ improvement [11].

We followed a standard operating procedure for both gait and cognitive evaluation. For gait evaluation, we used clinical measures, namely 10 m timed walk scores (assessing both steps and time), which is a simple, easy to administer and sufficiently validated procedure [27,28]. For cognitive evaluation, we used a standard neuropsychological battery, before and 48 h following tap test.

The cut-off values we used for the percentile improvement of gait are stricter than the majority of the reported in the literature [29,30,31] and were selected so that the responder status has a clinically meaningful importance. We decided to include the simultaneous improvement in MMSE and FAB in the criteria for defining positive response too, based on the guidelines of Mori et al. (2012) in order to include also patients that improve cognitively after LP in our investigation [23].

Our study revealed that the 10 m timed walk scores were significantly different before and 48 h after LP. Improvement of 10 m timed walk scores during tap test differed significantly between female and male patients. Females had a greater improvement both in steps and in time demanded to walk a 10 m distance. To the best of our knowledge this difference in tap test responsiveness between female and male patients with iNPH has not been described in the literature before. However, further studies are needed in order to shed more light on that gender-related difference.

As regards neuropsychological parameters, MMSE, FAB, delayed recall of 5WT and CLOX-2 differed significantly before and 48 h after LP. MMSE and FAB, for the evaluation of iNPH patients, have been previously used by Schmidt et al. in 2014 and Ko et al. in 2017, respectively [32,33], while CLOX and 5WT tests were used for the first time, to our knowledge, in order to assess iNPH patients. Nevertheless, in our study MMSE, FAB, delayed recall of 5WT and CLOX-2 proved to be sensitive in evaluating cognitive improvement during tap test.

In the questioning, as to whether any improvement should be (at whole or partially) attributed to a learning effect of this short time evaluation, Solana et al. in 2010 suggested that, in contrast to healthy controls, iNPH patients do not perform better in a repeating test setting of various neuropsychological tests without an intervention. Consequently, the improvement of neuropsychological tests’ score after an intervention, such as an evacuative LP is likely to reflect real clinical improvement rather than learning effect [34].

The total score of Radscale was similar between the tap test responders and non-responders. The same holds true for the individual sub-scores except for “callosal angle” sub-score, which was higher (meaning the angle is more acute) among tap test responders. This is in agreement with the findings of Onder et al. (2020) supporting that callosal angle, is an imaging marker, that could be used in patients with high suspicion of iNPH as an alternative of tap test to decide if they should have shunt surgery [12]. Lotan et al. in 2021 also suggested that callosal angle may be used as an imaging marker useful to predict tap test responsiveness [35]. Our results also support, Radscale’s “callosal angle” sub-score to be used as a specific indicator of patients that may respond to shunt surgery treatment. In contrast, total Radscale or other individual sub-scores appear to have limited value in this regard.

Pathophysiologically, it is not clear why the callosal angle may be narrower in tap test responders. In iNPH, it has been hypothesized that a vicious circle of a circulatory disturbance of the CNS and deceleration of its absorption leads to enlargement of the ventricles [3,36]. So, an impaired glymphatic transport might play a role [37]. This is also supported by studies in animal models [38]. Acute callosal angle could reflect the combination of elevation of dilated lateral ventricles and the restriction of the upper movement of the corpus callosum by the free margin of the falx cerebri [39].

We also found a strong positive correlation between absolute stroke volume and peak flow velocity. CSF stroke volume has been reported to be a more accurate marker than peak flow velocity regarding iNPH severity and larger stroke volumes have been associated with higher likelihood of shunt responsiveness [40,41]; however, the distributions of these two parameters were the same between tap test responders and non-responders in our study.

To the best of our knowledge, there is only one published prospective study, investigating possible correlations of Radscale with tap test outcome, which concluded that temporal horn measurement differs significantly between tap test responders and non-responders [31]. There were several limitations though in their study, such as the fact that the investigators evaluated only improvement, using Timed Up and Go (TUG) test before, and 24 h after the CSF tap test, while according to many studies the ideal time to evaluate any possible improvement during tap test is 48–72 h after LP [42,43]. Furthermore, no neuropsychological battery was used to investigate possible cognitive improvement after evacuative LP. Finally, the cut-off value for tap test responder status was stated relatively low (at 10% improvement) leading to a possible overestimation of the number of responders (83% in the study’s sample). There is, also, another retrospective study that clinico-radiologically investigated iNPH patients using Radscale among other scales. However, the study’s design and purpose were different, using only total score of Radscale and aiming basically to evaluate quantitative biomechanical analysis of gait and balance as a clinical and prognostic factor for iNPH patients [43].

Our study has certain limitations. Our cohort of patients lacks pathologic confirmation; however, this is an inherent disadvantage of such studies, and pathologic data in iNPH are generally lacking. Despite the above, the inclusion of patients was made according to well-established criteria. Another limitation may be the relatively small population (45 patients); however, this is sufficient for a single-center study. Larger multi-center studies should be conducted to further confirm these correlations. Quantitative human motion analysis using sophisticated equipment, such as accelerometers and instrumented 3d-video analysis of gait, were not implemented as our goal was to be dedicated in a more clinical approach. The lower rate of responders compared to other studies could be attributed to the strict response criteria implemented for movement and cognitive tests. Another limitation may be that evacuative LP was performed once, removing 30 to 50 mL of CSF. The results might have varied if we had removed 40–50 mL of CSF [44], or performed tap test on two consecutive days, removing 50 mL of CSF as described by Wikkelso et al. in 1986 [45]. Finally, our presented data are based on response to tap test, not shunting procedure, as we did not have “post-shunt” data of all patients. Thus, we cannot prove with certainty the prognostic value of Radscale and particularly callosal angle sub-score in patient selection for shunt surgery.

Our study aims to identify the imaging marker(s) that better associates with tap test responsiveness and consequently with greater possibility of good response to CSF shunting. In this way, its practical significance lies in improving the possibility of early recognition and more accurate diagnosis of iNPH patients and their better stratification according to their possibility of response to CSF shunting. This might help to better select patients that will be directed to CSF shunting, which is an invasive procedure. Until now, there is not any non-invasive established treatment for iNPH. Despite that, we should mention that there are studies in the current literature, conducted in animal models, regarding various non-surgical treatment options for hydrocephalus, some of which have initially positive outcomes [38].

More studies in the future may help to reach a consensus of generally accepted clinical scales and neuroimaging criteria leading to a better screening and diagnosis of patients with iNPH [46]. This will probably help iNPH patients to be better treated.

## 5. Conclusions

In conclusion, the results of the present study suggest that the total Radscale score does not differ between tap test responders and non-responders; however, a greater “callosal angle” sub-score, corresponding to a more acute angle, is associated with a positive tap test responder status and could be useful for the stratification of iNPH patients that will potentially respond to CSF shunting. However, more studies are needed, ideally including data from patients after shunt surgery.

## Figures and Tables

**Table 1:** Neuropsychological and gait data of studied patients.

N = 45	Neuropsychological						Gait	
	MMSE	FAB	5WT Immediate	5WT Delayed	CLOX-1	CLOX-2	10 m Timed Walk Test: Steps	10 m Timed Walk Test: Time
Before LP	24 (19–27.5)	10 (7–13)	5 (5–5)	4 (2–5)	8 (5–11)	10.5 (7–13)	22 (17–30.5)	12 (9.75–19.5)
48 h after LP	25 (19–29)	11 (8.5–14.5)	5 (4.75–5)	5 (3–5)	9 (5.5–12)	12 (8–13.5)	20 (17–29.5)	11.50 (8.25–18.5)
Median % change	0.042	0.125	0.007	0.073	0.022	0.057	−0.067	−0.136
*p*	0.001 ^†^	0.000 ^†^	NS ^†^	0.003 ^†^	NS ^†^	0.020 ^†^	0.004 ^†^	0.004 ^†^

N: total number of subjects, LP: lumbar puncture, 5WT: 5-word test, NS: non-significant, MMSE: mini-mental state examination, FAB: frontal assessment battery; neuropsychological and gait data are presented as median values (25th–75th percentile); ^†^ Wilcoxon Matched Pairs Test.

**Table 2:** Demographic and radiological characteristics of tap test responders and non-responders.

Variable	Tap Test Responders N = 19	Tap Test Non-Responders N = 26	*p*
Gender (F/M)	10/9	7/19	0.079 ^†^
Age	76 (71–79)	74 (69.8–78)	0.526 ^‡^
Grading scale	6 (5–7)	6 (4–7)	0.870 ^‡^
Disease duration (months)	36 (24–60)	24 (12–48)	0.225 ^‡^
Peak flow velocity	8.2 (5.7–10.6)	6.8 (4.6–11.9)	0.589 ^‡^
Absolute stroke volume	0.14 (0.11–0.32)	0.11 (0.05–0.36)	0.352 ^‡^
Evan’s index	2 (2–2)	2 (2–2)	0.169 ^‡^
Narrow sulci	1 (0–1)	1 (0–1)	0.214 ^‡^
Sylvian fissures	1 (0–1)	1 (1–1)	0.577 ^‡^
Focally enlarged sulci	0 (0–1)	0 (0–1)	0.202 ^‡^
Temporal horns	2 (2–2)	2 (1–2)	0.646 ^‡^
Callosal angle	1 (0–1)	0 (0–0.25)	0.036 ^‡^ *
Periventricular hyperintensity	2 (1–2)	2 (1–2)	0.522 ^‡^
Radscale total score	8 (7–9)	7 (7–8)	0.249 ^‡^

N: number of subjects; demographic and radiological parameters are presented as median values (25th–75th percentile); ^†^ χ^2^ test; ^‡^ Mann–Whitney U test; statistically significant *p*-values are indicated with a *.

**Table 3:** Neuropsychological and gait data of tap test responders.

N = 19	Neuropsychological						Gait	
	MMSE	FAB	5WT Immediate	5WT Delayed	CLOX-1	CLOX-2	10 m Timed Walk Test: Steps	10 m Timed Walk Test: Time
Before LP	21 (20–26)	9 (7–12)	5 (5–5)	4,5 (3–5)	7 (6–10)	10 (6.75–13)	25 (20–42)	13 (11–24)
48 h after LP	23 (21–28)	10 (9–13)	5 (5–5)	5 (4–5)	7,5 (5 -10.25)	11 (5.75–14)	21 (17–30)	12 (9–20)
Median % change	0.08	0.166	0.000	0.000	0.000	0.074	0.172	0.181
*p*	0.005 ^†^	0.001 ^†^	NS ^†^	NS ^†^	NS ^†^	NS ^†^	0.001 ^†^	0.005 ^†^

N: total number of subjects, LP: lumbar puncture, 5WT: 5-word test, NS: non-significant, MMSE: mini-mental state examination, FAB: frontal assessment battery; neuropsychological and gait data are presented as median values (25th–75th percentile); ^†^ Wilcoxon Matched Pairs Test.

**Table 4:** Neuropsychological and gait data of tap test non-responders.

N = 26	Neuropsychological						Gait	
	MMSE	FAB	5WT Immediate	5WT Delayed	CLOX-1	CLOX-2	10 m Timed Walk Test: Steps	10 m Timed Walk Test: Time
Before LP	25.5 (18.75–28)	11.5 (6.75–15)	5 (4–5)	4 (1–5)	9 (4–12.75)	11 (7.5–13)	19 (16–29.25)	10.75 (9–16.25)
48 h after LP	27 (17.5–29)	13 (7.75–15.25)	5 (4–5)	5 (2–5)	10 (6–14)	12 (9–13)	19 (15.75–28.5)	10.25 (7.5–18.25)
Median % change	0.0345	0.068	0.000	0.000	0.000	0.000	0.015	0.057
*p*	0.012 ^†^	NS ^†^	NS ^†^	NS ^†^	NS ^†^	NS ^†^	NS ^†^	NS ^†^

N: total number of subjects, LP: lumbar puncture, 5WT: 5-words test, NS: non-significant, MMSE: mini-mental state examination, FAB: frontal assessment battery; neuropsychological and gait data are presented as median values (25th–75th percentile); ^†^ Wilcoxon Matched Pairs Test.

## Data Availability

The data presented in this study are available upon reasonable request from the corresponding author. The data are not publicly available due to privacy restrictions.

## Supplementary Materials

The following supporting information can be downloaded at: https://www.mdpi.com/article/10.3390/jcm11102898/s1, Figure S1: Significant correlations among clinical and imaging parameters before Bonferroni correction.; Figure S2: Significant positive correlation of absolute stroke volume with absolute peak flow velocity of CSF; Table S1: Median percentile improvement of the parameters used on the implemented criteria for positive tap test responder status, among responders and non-responders.
