# Supplementary material for: Callosal Angle Sub-Score of the Radscale in Patients with Idiopathic Normal Pressure Hydrocephalus Is Associated with Positive Tap Test Response
Source: J Clin Med. 2022 May 20;11(10):2898. doi: 10.3390/jcm11102898 (PMC9143138; doi:10.3390/jcm11102898)
Supplement: Supplementary file 1 [file jcm-11-02898-s001.zip › jcm-1711446-supplementary.pdf]

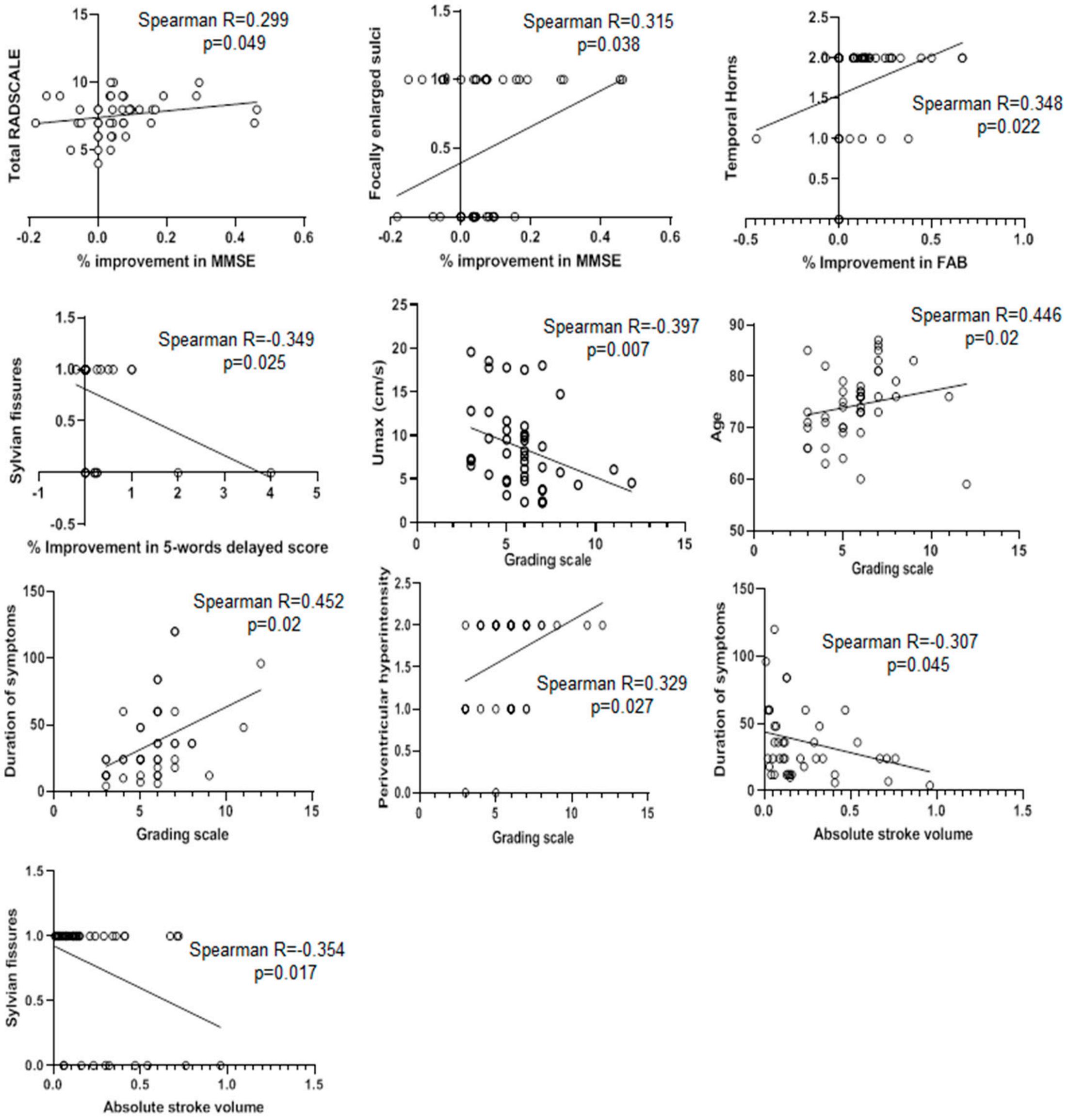

Supplementary Figure S1. Significant correlations among clinical and imaging parameters before Bonferroni correction. Significant positive correlations between iNPH Grading Scale with age of patients (spearman R=0.446, p=0.02), duration of symptoms (spearman R=0.452, p=0.02), and "periventricular hyperintensity" sub-score in brain MRI (spearman R=0.329, p=0.027), and a significant negative correlation between iNPH Grading Scale and absolute peak flow velocity (spearman R= -0.397, p=0.007). Percentile improvement in FAB score following TAP test had a significant positive correlation with Temporal Horns score (spearman R=0.348, p=0.022). Percentile improvement in 5-words delayed score during TAP test had a negative significant correlation with "Sylvian fissures" sub-score (spearman R= -0.349, p=0.022). Absolute stroke volume had negative significant correlation with the duration of the disease (spearman R= -0,307, p=0.045) and the "Sylvian fissures" sub-score (spearman R= -0,354, p=0.017).

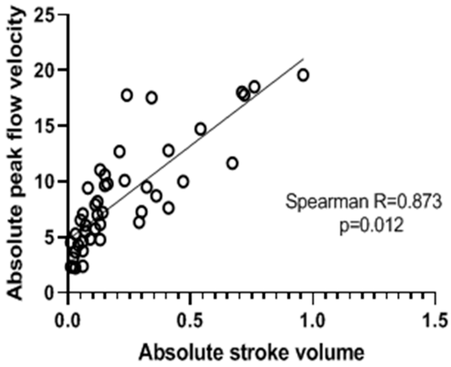

Supplementary Figure S2. Significant positive correlation of absolute stroke volume with absolute peak flow velocity of CSF.

Supplementary Table S1. Median percentile improvement of the parameters used on the implemented criteria for positive Tap test responder status, among responders and non-responders.

Parameter	Median percentile improvement during Tap test	
	**Tap test responders**	**Tap test non-responders**
MMSE	8%	3.5%
FAB	16.6%	6.8%
10-meter timed walk test: steps	17.2%	1.5%
10-meter timed walk test: time	18.1%	5.7%

MMSE: MiniMental State Examination, FAB: Frontal Assessment Battery
